# Curriculum Innovation: Neurocritical Care EEG Rounds: A Model to Improve Neurology Resident EEG Interpretation

**DOI:** 10.1212/NE9.0000000000200301

**Published:** 2026-04-01

**Authors:** Ellen Sylvie Sanchez Mas, Mitchell Lloyd Powell, Marco Malaga, Corey Elam Goldsmith, Rahul Damani, Lu Lin

**Affiliations:** 1 Department of Neurology, Baylor College of Medicine, Houston, TX;; 2 Section of Neurophysiology, Department of Neurology, Baylor College of Medicine, Houston, TX; and; 3 Section of Neurocritical Care and Vascular Neurology, Department of Neurology, Neurosurgery & Center for Space Medicine, Baylor College of Medicine, Houston, TX.

## Abstract

**Background and Objectives:**

Continuous EEG (cEEG) has emerged as an important monitoring tool in the intensive care unit (ICU). Traditional EEG curricula for neurology residents mainly consist of didactic lectures, which often yield limited exposure to ICU EEG concepts and are insufficient to develop proficiency. The aim of this study was to develop a curriculum to enhance neurology resident ICU EEG education. The objectives included: (1) improving residents' ICU EEG knowledge by providing exposure to real ICU EEG cases and (2) facilitating discussion of EEG findings and monitoring plans within the multidisciplinary team.

**Methods:**

We developed and implemented Neurocritical Care (NCC)-EEG rounds, a daily 30-minute virtual conference targeting neurology residents during their NCC rotation, to review cEEG studies of patients under NCC care. Residents' comfort and knowledge toward ICU EEG interpretation were assessed before and after 4-week NCC rotation using self-designed questionnaires. An acceptability survey was distributed to residents and the neurophysiology team at the conclusion of the study reporting period to gather feedback. The paired *t* test was used to analyze the pretest and posttest data.

**Results:**

Data from 20 residents showed significant improvement in subjective comfort (5-point Likert) with overall EEG interpretation (+0.6, [95% CI 0.22–0.98], *p* = 0.0040), identifying epileptic seizures (+0.45, [0.09–0.81], *p* = 0.0158), identifying artifacts (+0.45, [0.03–0.87], *p* = 0.0351), classifying EEG backgrounds (+0.45, [0.09–0.81], *p* = 0.0158), and mean total objective knowledge scores (6.00 ± 2.43 vs 4.65 ± 2.6, [mean ± SD, maximum score 10], *p* = 0.0226) after their NCC rotation. The acceptability survey (5-point Likert, mean ± SD) showed that both neurology residents (N = 7) and neurophysiology attendings/fellows (N = 6) found the model to be effective as a teaching (4.3 ± 0.7 and 4.3 ± 0.7, respectively) and communication (4.7 ± 0.5 and 4.5 ± 0.8, respectively) tool.

**Discussion:**

Our model of daily NCC-EEG rounds integrates case-based EEG review into neurology residents' daily clinical care. It is shown to be an effective strategy in improving neurology residents' ICU EEG education and strengthening interteam communication at our institution. Further work is needed to assess the model's long-term effectiveness and generalizability.

## Introduction

Continuous EEG (cEEG) provides real-time monitoring of cerebral activity and is widely used in critically ill patients to diagnose seizures and status epilepticus, assess the level of consciousness, identify cerebral ischemia, and predict prognosis after cardiac arrest.^1,2^ Its use in the intensive care unit (ICU) has increased significantly, with data showing more than a 10-fold increase in cEEG use from 2004 to 2013 in the United States (US).^3^ However, even in tertiary care medical centers, delays may occur in EEG acquisition and interpretation by EEG readers.^4^ Thus, educating the clinical care team, including neurology residents, to identify common ICU EEG findings may bridge this gap, leading to timelier decision making in patient care.

Proficiency in EEG reading is included as a graduation milestone for neurology residents in the Accreditation Council for Graduate Medical Education guidelines, but only around 60% of final year residents reported feeling confident in their EEG reading skills.^5^ This may at least be partly due to the short duration of EEG exposure during training. In the United States, a typical neurology resident, on average, completes only 1.5–1.7 months of EEG rotations, and the number of EEGs read during the rotations is variable.^6^ A national survey of neurology residency program directors on EEG education revealed that the main perceived barriers in resident EEG education were ineffective didactics and insufficient EEG exposure. Additional hurdles included demanding clinical workload and limited time for education. Resident exposure to ICU EEG was not investigated in this survey.^7^ Similarly, residents reported in a survey that the most common education obstacles were not enough exposure to EEG, insufficient responsibility to read the studies, and inability to associate EEG concepts to direct patient care.^8^

Traditional interventions for ICU EEG education consist of formal didactics, typically of short duration, with learning outcomes evaluated only in the short term. One study found that after completing a face-to-face EEG course followed by multiple e-learning sessions, 71% of participants were able to accurately recognize basic ICU EEG patterns.^9^ An online educational module designed for critical care fellows was also shown to improve EEG knowledge.^10^ While these approaches provide a solid foundational understanding, their implementation can be challenging due to residents' demanding clinical workloads and limited time. In addition, it is often difficult for residents to apply newly acquired EEG knowledge to real-time patient care. Only 3 of 29 studies included in a meta-analysis about EEG education in the critical care setting included neurology residents.^11^ Previous studies with anesthesia residents demonstrated greater efficacy in ICU EEG learning after weekly^12^ and biweekly^13^ 1:1 sessions with a neurophysiologist, compared with a traditional didactic approach, but did not address clinical management or interdisciplinary team communication as objectives. These findings highlight the importance of propagating novel interventions for long-term and sustained ICU EEG teaching for neurology residents.

## Objectives

Our goal was to produce a curriculum that would enhance ICU EEG education for neurology residents in our institution. The objectives of the curriculum included: (1) improving residents' ICU EEG knowledge by providing exposure to real ICU EEG cases and (2) facilitating discussion of EEG findings and monitoring plans within the multidisciplinary team. We developed a case-based EEG teaching model that was implemented during residents' neurocritical care (NCC) rotation, enabling residents to learn relevant ICU EEG knowledge from real cases and integrating EEG education with patient care. We also anticipated that this model would promote a formal and consistent avenue of discussion for patient management decisions between the NCC and neurophysiology teams.

## Methods

### Curriculum Development

We developed and implemented a daily Neurocritical Care (NCC)-EEG Rounds for neurology residents during their NCC rotation. A 30-minute video conference session was conducted on weekdays at 3:30 pm local time. The time was chosen by the NCC and neurophysiology teams to minimize disruption to patient care and allow the EEG readers sufficient time to finish reviewing and placing reports. Participants invited to attend included NCC team members (rotating adult and child neurology residents, NCC fellows, and NCC attending) and neurophysiology team members (neurophysiology technologists, neurophysiology fellow, and neurophysiology attending). Each NCC-EEG round began with residents presenting a brief clinical history and the reason for EEG for their own patients on cEEG monitoring, followed by EEG review led by the neurophysiology fellow/neurophysiology attending with residents participation, and concluded with discussion of clinical management plans between the NCC and neurophysiology teams (e.g., treatment, the length of monitoring). The primary focus was to educate residents on various ICU EEG patterns. This format was chosen to maximize direct EEG exposure and foster interactive discussion, particularly between residents and EEG readers.

To encourage participation, the adult neurology chief resident distributed an email to residents a week before their NCC rotation, introducing the format of the NCC-EEG rounds and including the pretest questionnaire. The neurophysiology team sent a daily reminder about the round to the NCC team through an electronic messaging platform.

### Study Setting and Participants

The study was conducted at Baylor St. Luke's Medical Center, a tertiary medical center with more than 2,000 inpatient EEG studies performed annually. The daily NCC-EEG rounds were initiated in July 2024 and available for all adult neurology post-graduate year 1 (PGY1), PGY2, or PGY3 and child neurology residents rotating on their 4-week NCC rotation. Assessment data collected between July 2024 and April 2025 were included in the data analysis. Any residents who did not complete both pretest and posttest questionnaires were excluded from the analysis. The acceptability survey was distributed to residents and the neurophysiology team in May 2025.

### Assessments

To assess subjective and objective improvement in residents' ICU EEG skills, a self-designed questionnaire was given at the start and end of their 4-week NCC rotation. Each pretest and posttest consisted of 4 subjective “comfort” items (Kirkpatrick level 1) and 10 objective “knowledge” items (Kirkpatrick level 2). The 4 items to report subjective comfort level regarding various aspects of EEG interpretation were identical on the pretest and posttest. Ten objective “knowledge” items included 9 questions with a fictional case description and a single-page EEG image testing 1 unique ICU EEG pattern (different case description and EEG images on the pretest and posttest) and 1 multiple-choice question about the definition of seizures.^14^ (Pretest and posttest questionnaires included as eAppendix 1). A 14-item questionnaire was selected as the minimum number of items to assess residents' comfort and knowledge while balancing the length of the questionnaire to retain maximal resident involvement. EEG images used in the questionnaires were deidentified from prior patient cases at our institution, selected based on formal EEG reports describing specific EEG patterns, then reviewed and confirmed by 1 neurophysiology attending. The questionnaires were developed by 1 neurophysiology fellow and 1 neurophysiology attending and then pilot-tested in 2 neurology residents (excluded from the study participation) to ensure clarity of the questions and answer keys. Completion of the questionnaires was encouraged by the residency program director and study authors but was not considered mandatory. Consent to participate in the data analysis was considered implicit with completion of the questionnaires.

### Acceptability Survey

A survey was distributed at the conclusion of the reporting period for this study to gather feedback from neurology residents and neurophysiology attendings/fellows regarding their reaction to the daily NCC-EEG rounds (Kirkpatrick level 1). Each survey consisted of an item inquiring about the average attendance, items on a 5-point Likert scale inquiring the usefulness of the rounds as an education tool and an interteam communication tool, as well as free-response items to provide open-ended feedback (Survey questionnaires included as eAppendix 2). Completion of the surveys was encouraged but was not considered mandatory. Consent to participate in the data analysis was considered implicit with completion of the surveys.

### Data Analysis

Subjective and objective portions of the pretest and posttest questionnaires were analyzed separately. Normality of the data was confirmed analytically with the Kolmogorov-Smirnov and Shapiro-Wilk tests. Pretest and posttest data were analyzed using paired *t* test and considered statistically significant at *p* < 0.05, confidence interval was calculated to determine precision, and Cohen *d* was calculated to determine effect size. Acceptability survey data were presented as mean ± standard deviation or qualitatively.

### Standard Protocol Approvals, Registrations, and Patient Consents

This project was determined by Baylor College of Medicine's Institutional Review Board (IRB) as an education quality improvement activity and did not require IRB approval. Participation in the daily NCC EEG rounds and pretest and posttest questionnaires was encouraged but not mandatory. All resident data were deidentified for the process of data analysis, discussion, and publication. The authors of this manuscript are not aware of any conflicts of interest.

### Data Availability

Data not provided in this article because of space limitations may be shared (anonymized) at the request of any qualified investigator for purposes of replicating procedures and results.

## Results

### Participants

A total of 32 residents participated in the curriculum during the study report period. Twenty residents completed both the pretests and posttests, thus were included in the data analysis. The residents consisted of 18 adult neurology (4 PGY1s, 8 PGY2s, 6 PGY3s) and 2 child neurology residents. Nine (45%) of them were female, and 11 (55%) were male. The mean number of days between completion of pretest and posttest was 27.4 ± 9.4.

### Assessment

**Figure 1:**
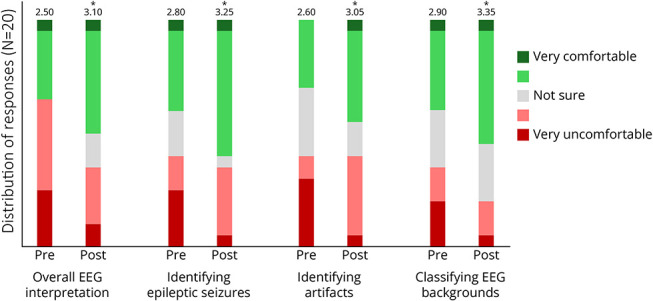
Change in Subjective Comfort Level of EEG Interpretation (“Comfort” Score) Resident comfort level to various EEG interpretation aspects was reported on 5-point Likert scale on the pretest and posttest. Mean “comfort” score of all subjects (N = 20) was shown. Asterisk (*) denotes *p* < 0.05 using the paired *t* test.

Each subjective item used the 5-point Likert scale and was scored numerically from 1 to 5. Residents reported significantly higher mean “comfort” scores on the posttest compared with the pretest on all 4 items: overall EEG interpretation (+0.6, [95% CI 0.22–0.98], *p* = 0.0040), identifying epileptic seizures (+0.45, [0.09–0.81], *p* = 0.0158), identifying artifacts (+0.45, [0.03–0.87], *p* = 0.0351), and classifying EEG backgrounds (+0.45, [0.09–0.81], *p* = 0.0158) (Figure 1 and Table).

**Table:** Statistical Analysis of All Subjective “Comfort” and Objective “Knowledge” Items

	Pretest	Posttest	Difference	95% CI	*p* Value	Cohen *d*
Subjective “Comfort” Score						
Overall EEG interpretation	2.50	3.10	+0.60	0.22–0.98	0.0040^a^	0.48
Identifying epileptic seizure	2.80	3.25	+0.45	0.09–0.81	0.0158^a^	0.37
Identifying artifacts	2.60	3.05	+0.45	0.03–0.87	0.0351^a^	0.39
Classifying EEG background	2.90	3.35	+0.45	0.09–0.81	0.0158^a^	0.40
Objective “Knowledge” Score						
Focal slowing	15	55	+40	12–68	0.0075^a^	0.90
Lateralized periodic discharges	35	70	+35	8–62	0.0153^a^	0.73
Mild encephalopathy	35	65	+30	3–57	0.0298^a^	0.61
Suppression	65	85	+20	−9 to 49	0.1625	0.46
Seizure	50	65	+15	−8 to 38	0.1864	0.30
Breach rhythm	35	40	+5	−23 to 33	0.7157	0.10
Burst suppression	55	55	0	−26 to 26	1.00	0
Moderate encephalopathy	55	55	0	−30 to 30	1.00	0
Myoclonic status epilepticus	45	45	0	−34 to 34	1.00	0
Generalized periodic discharges	75	65	−10	−40 to 20	0.4936	0.21

Test scores are presented as mean for “Comfort” Score (maximum score of 5.0 per item) and percentage correct (%) for “Knowledge” Score. N = 20.

a Denotes *p* < 0.05 using the paired *t* test.

**Figure 2:**
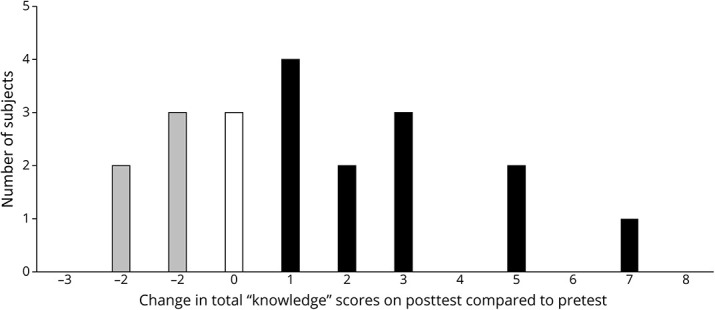
Change in Objective Measures of EEG Interpretation (Total “Knowledge” Score) Graph showed number of subjects with corresponding change in total score. Twelve of 20 subjects showed improvement (change of +1 or higher). Mean of pretest score: 4.65 ± 2.6 vs posttest score: 6.00 ± 2.43. *p* = 0.0226 (paired *t* test).

Each objective item used a multiple-choice system with 1 correct answer (1 point for each correct answer, maximum 10 points). Residents earned significantly higher total “knowledge” scores on the posttest compared with the pretest (6.00 ± 2.43 vs 4.65 ± 2.6, *p* = 0.0226) (Figure 2). Analysis of performance on individual EEG concepts showed that significantly higher percentage of residents provided correct answers on posttest compared with pretest on 3 items: focal slowing (+40%, [12.0–68.0], *p* = 0.0075), lateralized periodic discharges (+35%, [7.5–62.5], *p* = 0.0153), and mild encephalopathy (+30%, [3.3–56.7], *p* = 0.0298). The remaining 7 items did not show a statistically significant change (Table).

### Survey Evaluation

At the conclusion of the study reporting period, an acceptability survey was sent to the 20 neurology residents who completed both the pretest and posttest, as well as total 8 neurophysiology team members (5 attendings and 3 fellows).

Seven residents responded to the survey (response rate: 35%) and reported that they attended 3.7 ± 0.9 (mean ± SD) out of 5 rounds per week on average. On a scale of 1 (never) to 5 (always), residents indicated that the rounds time was protected most of the time (4.0 ± 0.5). They found rounds to be useful for NCC and neurophysiology team communication (4.7 ± 0.5), useful for their EEG education (4.3 ± 0.7), and digestible even for junior residents (3.9 ± 0.8). On the free-response items, residents reported that the aspects they liked the most about this model were team communication and correlating clinical findings with EEG. The most commonly reported factors preventing them from attending sessions were afternoon patient care commitments (e.g., admissions, clinic, and stroke codes). Residents also suggested that timing of the sessions could be optimized and a more structured curriculum for juniors may be beneficial.

Four neurophysiology attendings and 2 neurophysiology fellows responded to their survey (response rate: 75%). Four respondents indicated that sessions took place 51%–75% of the time, and 2 respondents indicated that sessions took place 76%–100% of the time. On a scale of 1 (never) to 5 (always), neurophysiology fellows/attendings indicated that they found the rounds to be useful for interteam communication (4.5 ± 0.8) and for resident education (4.3 ± 0.7). They also reported in the free-response session that the rounds were great for improving patient care, explaining monitoring goals, and reducing unnecessary monitoring.

## Discussion

A recent scoping review on EEG instruction reveals a trend toward multimodal approaches, integrating traditional didactic instruction with online-based instruction and hands-on experiential learning.^15^ To enhance neurology resident ICU EEG education, we developed and implemented a novel EEG education curriculum—daily NCC-EEG rounds at our institution. We assessed its impact on neurology residents' level of comfort and knowledge about ICU EEG over the course of 4 weeks. At the end of the 4-week period, we observed a statistically significant improvement in both subjective comfort level and objective knowledge scores among a group of 20 residents. In addition, feedback from neurology residents and neurophysiology attendings/fellows was supportive that this model was effective in promoting resident education and strengthening communication between clinical care teams.

Our model addresses the challenges in EEG education of neurology residents while emphasizing ICU EEG knowledge by being a practical and clinical case-based approach. Our design fosters an interdisciplinary review and discussion of the EEGs of the same patients that neurology residents are taking care of during their NCC rotation. The acceptability survey results support that this model is feasible to conduct. This interactive virtual session not only improves resident ICU EEG knowledge but may also make interdisciplinary discussion between different clinical care teams more efficient and nuanced. Poor communication has been identified as one of the antecedents of human errors in the ICU, which affects patient safety and quality of care.^16^ In our study, those who responded to the acceptability survey reported subjective improvement in interteam communication. Hopefully, the downstream effect of this practice will be to reduce time to medical intervention in cases of epileptic emergencies, reduce unnecessary monitoring, distribute electrophysiologic resources more appropriately, and generally improve patient outcomes.

Data from previous studies on EEG education for neurology residents revealed that the most challenging EEG findings include sedative effects (e.g., drug-induced spindles), focal epileptogenic potentials, and focal slowing.^6^ Similarly, our pretest identified that the least known patterns amongst our residents were focal slowing, mild encephalopathy, lateralized periodic discharges, and breach artifact. Significant improvement was seen on the posttest in identifying 3 of these 4 patterns. A prospective multicenter study assessing the impact of a critical care EEG curriculum in ICU staff including residents found that reactivity, periodic patterns, and burst suppression were the most difficult concepts to learn.^9^ This was similar to our results, in which the concepts of generalized periodic discharges, moderate encephalopathy, burst suppression and myoclonic status did not show improvement on the posttest. An alternative hypothesis for why certain concepts showed improvement, while others did not is uneven exposure to different topics during the rounds. Our data collection did not include a tabulation of which topics were covered during each session. Finally, our test questionnaire only contains single-page EEG images, which might make it challenging to identify certain patterns (e.g., burst suppression).

Our study has several limitations. First, as an initial pilot study of the daily NCC-EEG rounds model, it is restricted to a single center and there are no extended data points to evaluate sustained improvement. Its generalizability is also limited to those institutions with dedicated neurocritical care and neurophysiology teams. Second, there was significant attrition seen in the number of residents who completed both the pretests and posttests (62.5%) and the acceptability survey (response rate 35%), which may have biased the results toward those more motivated or participated more in the rounds. The attrition can be in part due to the voluntary nature of participation, as this was the first pilot period, participation was encouraged but not required as part of the curriculum. After this initial pilot period was completed, it is now integrated into resident NCC rotation curriculum, with the expectation for residents to participate and complete the questionnaires. It would be interesting to see whether the effect of the model persists after this change. Furthermore, the timing of the survey may contribute to recall bias among those who completed NCC rotation several months earlier. Finally, to maximize the available sample size, we chose not to designate a control group. Validation of the questionnaires was also limited to expert consensus, due to concerns about limiting questionnaire length to minimize attrition. Ideally, each concept should be tested multiple times to increase internal validity of the questionnaires.

Our results should be interpreted with caution. Resident attendance proved difficult to ensure, as residents were not always able to attend all NCC-EEG rounds due to urgent clinical duties in the ICU. Although residents reported in the survey that they attended 3.7 ± 0.9 out of 5 sessions per week, data regarding actual attendance were not kept. In addition, we did not collect data about EEG concepts discussed at each session (dictated by the patient cases present in the ICU), which may have an undue influence on which topics the residents became more comfortable with. Collection of such data in the future would allow us to correlate frequency of teaching more directly with improvement in resident comfort and knowledge about specific topics. It can also help improve the curriculum, by directing the neurophysiology team to add or focus on underrepresented concepts. Our acceptability survey was enlightening in that it showed the perceived value of the daily NCC-EEG rounds was high, as an effective tool in teaching and conduit for interdisciplinary team communication. However, we obtained no objective data to confirm those perceptions. A large area of untapped data mining includes metrics that investigate the model's effectiveness in improving NCC neurophysiology communication and patient care, by tracking appropriate vs unnecessary cEEG monitoring, overall cEEG monitoring time, time to detection and reporting of concerning EEG patterns, time to medical intervention, and associated changes in long-term clinical outcomes.

Future studies will be directed toward focused teaching on weak topics identified through the pretest and using a tailored teaching approach based on resident knowledge level. As discussed, resident attendance and topics discussed can be tabulated, as well as further metrics investigating the model's effectiveness in improving interteam communication and outcomes in patient management. In addition, this model could be further expanded to include other audiences in the pretest and posttest analysis (e.g., NCC fellows and neurophysiology technologists).

Our study showed that neurology residents' subjective comfort and objective knowledge in ICU EEG pattern recognition increased after implementation of daily NCC EEG rounds. Acceptability survey data from participants also suggested that this model is effective in strengthening communication between the NCC and neurophysiology teams. Our model integrates a case-based EEG curriculum into residents' daily clinical care, addressing a critical gap in resident EEG teaching by providing regular exposure to relevant ICU EEG cases in a digestible format. While plenty of work remains to collect the rigorous data necessary to demonstrate the daily NCC EEG rounds' effectiveness over time, especially relating to neuromonitoring quality metrics, our model offers an easily implementable innovation that other institutions may adopt and build on.

## Glossary

cEEG: continuous EEG
ICU: intensive care unit
IRB: institutional review board
NCC: neurocritical care
PGY: post-graduate year
